# Exposure assessment to inhalable and respirable dust in the post — earthquake construction sites in the city of l'Aquila

**DOI:** 10.1002/1348-9585.12296

**Published:** 2021-12-01

**Authors:** Riccardo Mastrantonio, Angela Civisca, Eugenio Siciliano, Enrica Inglese, Tamara Lippolis, Domenico Pompei, Lucio Cococcetta, Maria Scatigna, Leila Fabiani

**Affiliations:** ^1^ Department of Life, Health & Environmental Sciences University of L'Aquila L'Aquila Italy; ^2^ Regional Directorate of Abruzzo National Institute for Insurance Against Industrial Injuries L’Aquila Italy; ^3^ Local Health Authority of Avezzano‐Sulmona‐L'Aquila L'Aquila Italy; ^4^ Ente scuola edile – Comitato paritetico territoriale L'Aquila Italy

**Keywords:** chemical risk assessment, chemical risk construction industry, dust exposure construction industry, occupational exposure, silica dust exposure

## Abstract

**Objectives:**

Following an earthquake that occurred in middle Italy in 2009, the involved territory hosted in 10 years thousands of construction sites. The aim of this study is to assess inhalable, respirable, and respirable crystalline silica exposure of the construction workers involved in the rebuilding activities.

**Methods:**

Six construction companies joined the study and hosted the air sampling activities. We identified four work tasks: bricklayer and similar; scaffolder and carpenter; manual demolition; other tasks. We reported 8‐h time‐weighted concentrations.

**Results:**

The “All tasks” geometric mean concentration of inhalable dust was 4.73 mg/m^3^ and the higher, TLV exceeding exposure was observed for “Manual demolition workers” (13.92 mg/m^3^, GM). The “All tasks” geometric mean concentration of respirable dust was 0.25 mg/m^3^ and no TLV‐exceeding exposure (geometric mean values) was observed among the work‐related groups. About the respirable crystalline silica dust exposure, the “All tasks” average concentration was 0.004 mg/m^3^. No TLV‐exceeding exposure was observed among the whole data sample.

**Conclusions:**

The comparison of the results shows that manual demolition workers are exposed to high levels of inhalable dust, exceeding the TLV‐TWA (Threshold Limit Values‐Time Weighted Average) limit of 10 mg/m^3^. About the respirable dust concentration, none of the analyzed work task dust concentrations exceeded the TLV‐TWA limit of 3 mg/m^3^ (geometric mean values). Measurements of respirable crystalline silica dust have shown levels below the threshold limit value of 0.025 mg/m^3^. Our findings overall match with the available scientific data.

## 1 INTRODUCTION

Following the 2009 earthquake, 8124 construction sites arose in the city of L'Aquila and the surrounding area. Of these, 592 construction sites are currently in place.^1^

Construction site activities involve significant risks and hazards associated with chemical exposure; this exposure is due to work processes and the generation or use of substances and mixtures. Because of this, workers in construction industries are commonly exposed to fibers and dusts with variable aerodynamic particle size distributions, particularly workers employed in building demolition sites.^2^, ^3^, ^4^ The purpose of this study is to determine personal dust exposure in the construction sites of the city of L'Aquila.

The construction industry is a huge economic sector that involves several jobs and work tasks. Thus, it can be difficult to assess or quantify accurately dust exposure levels among construction workers.^5^ Dust exposure may cause respiratory diseases such as chronic obstructive pulmonary disease (COPD).^5^, ^6^

In our study, we considered only the inhalable and respirable particle fractions, defined as follows:

- Inhalable fraction: Mass fraction of total airborne particles which is inhaled through the nose and mouth with a 50% cut‐point of 100 µm;
- Respirable fraction: Mass fraction of inhaled particles which penetrate to the unciliated airways with a 50% cut‐point of 4 µm.^7^, ^8^

Some crystalline silica particles are classified as respirable crystalline silica (RCS) due to their granulometry and whose inhalation may cause severe pulmonary diseases. Crystalline silica is widely used in construction sites and is a natural mineral found in rocks, sand, clay, and gravel.^9^

Crystalline silica particles smaller than 10 µm in aerodynamic diameter are biologically active and can, therefore, penetrate into the lung causing silicosis and other diseases, especially if silica is freshly fractured.^10^, ^11^ Exposure to RCS in the construction industry is generally higher than the occupational exposure levels (OEL).^12^ RCS has been classified by the International Agency for Research on Cancer (IARC) as a Group 1 carcinogen.^13^ Furthermore, evidence indicates that low cumulative exposure to RCS increases the incidence of lung diseases, such as COPD, bronchitis, emphysema, and kidney diseases.^6^, ^13^, ^14^

The first aim of this study is to estimate the personal exposure to inhalable and respirable dust in post‐earthquake construction sites. Our measurements have been compared with the occupational exposure levels known as threshold limit values (TLVs), issued by the ACGIH (American Conference of Governmental Industrial Hygienists). TLVs refer to the airborne particulate concentration, which is set at 10 mg/m^3^ for inhalable dusts and at 3 mg/m^3^ for respirable dust. These threshold limit values, defined as a time‐weighted average (TWA), can be applied for a conventional 8‐h workday and a 40‐h workweek in which nearly all workers may be repeatedly exposed for a working lifetime without showing adverse effects. A further aim of this study is to investigate the exposure to RCS; for this carcinogenic pollutant, ACGIH recommends a TLV‐TWA of 0.025 mg/m^3^.^15^

ACGIH proposes TLVs as safe as possible for human health. For completeness, we also report the TWA limit values recommended by the Occupational Safety and Health Administration (OSHA) and by the National Institute for Occupational Safety and Health (NIOSH). The two Agencies recommend the permissible exposure limits (PEL) and the recommended exposure limits (REL), respectively. The PEL are defined as the maximum airborne TWA concentration that should not be exceeded at any time during an 8‐h workday and a 40‐h workweek. The REL are defined as the maximum TWA concentration that should not be exceeded during a 10‐h workday and a 40‐h workweek.^16^ These limits are summarized in Table 1. We report these American OELs because Italian regulations do not supply any.

**TABLE 1:** Occupational Exposure Limit Values (OELVs) for RCS, inhalable and respirable dust from different agencies

	OSHA PEL	NIOSH REL	ACGIH TLV
TWA (Time Weighted Average) RCS	0.05 mg/m^3^	0.05 mg/m^3^	0.025 mg/m^3^
TWA (Time Weighted Average) respirable dust	5 mg/m^3^	N.P.	3 mg/m^3^
TWA (Time Weighted Average) inhalable dust	15 mg/m^3^	N.P.	10 mg/m^3^

## 2 METHODS

The most damaged Italian region was Abruzzo (the earthquake hit 56 municipalities) but the seismic crater extended also to Marche, Lazio, and Umbria. Our study has been conducted in the province of L'Aquila which was the most damaged, especially in the city center. The size of the yards was variable because we included in the study building sites located within:

1. The center of the city and of the neighboring municipalities (mainly historical buildings).
2. The suburb (mainly private dwellings).

However, the yards were not bigger than a five‐story building.

Since the participation to the project was voluntary, the personnel of the Local Health Authority, the Joint Local Committee, and the University reached out to the building companies through informal channels (verbally or by telephone). The University and the Joint Local Committee invitation was based on existing professional relationships. The local health authority had a telephonic communication with 51 companies, chosen among those with at least one active building site.

To date, six companies have joined the project and the sampling activities in the construction sites have been carried out after a survey of the most relevant work tasks.

For each yard, we found a variable number of workers (arithmetic mean: 11.82; geometric mean 7.49; minimum 2; maximum 50). We often found workers employed by subcontracting companies that we could not involve in the sampling activities. The selection of the workers for sampling was conditioned by the ongoing working activities. Our aim was to assess the dust exposure without modifying the production cycles; in light of this, we prioritized the investigation of the exposure of bricklaying, manual demolition, and scaffolding/carpentry activities (the activities are fully explained in the next sentences of this section). In the absence of consensual workers or in the absence of these activities, we applied our personal samplers on workers engaged in the activities we listed in the “other work tasks activities”.

In order to perform personal indoor air sampling, the selection of the workers was carried out based on the work tasks within the building site. There were four selected work tasks: “bricklayer or similar”, “scaffolder and carpenter”, “manual demolition worker” and “other work tasks”; the latter category includes a variety of activities related to different job types. Each of the above‐mentioned tasks involve the performance of various duties. The following are the specific tasks identified in the construction sites we visited to perform the sampling:

1. Bricklayer and similar: masonry work, resin and mortar injections, insulation, plasterwork, roofing work, installation of systems;
2. Scaffolder and carpenter: Carpentry activities and scaffolding erection.
3. Manual demolition worker: Demolition works and buried electrical conduits.
4. Other tasks: Installation of window and door moldings, drilling, wood finishing, woodcutting, electrical wiring, driver, sandblasting, installation of plumbing systems, and welding.

The inhalable and respirable indoor dust measurements were not taken in parallel on the same worker. Table 2 shows the number of companies that joined the study, the measurements performed, the number of workers, and the total number of sampling hours (excluding breaks). In view of the voluntariness of the participation and taking into account the ongoing working activities of each specific construction site, the number of samples stratified on the four groups are different. The goal of the study is “taking a picture” of the dust exposure in the post‐earthquake rebuilding yards.

**TABLE 2:** Sampling duration in hours and workers involved by dust diameter

	Number of companies	Number of construction sites	Number of measurements	Number of workers	Total sampling hours
Inhalable dust	6	28	47	47	265
Respirable dust	5	28	44	43	243

Personal air sampling was performed using the following sampling train:

1. SKC AirCheck XR5000 sample pumps.^17^
2. 10‐mm diameter silicone tubing.
3. SKC I.O.M. particle size‐selective sampler for inhalable dust.^18^
4. SKC GS‐3 cyclone for respirable dust.^19^

According to the NIOSH Manual of the Analytical Methods,^20^ the World Health Organization,^21^ and the ISO 7708/1995,^8^ the ideal sampler for the respirable convention should have a 50% cut‐point of 4 µm. For the inhalable convention, the sampler should have a 50% cut‐point of 100 µm.

All the sample collection substrates were 25‐mm PVC filters with 5 µm pore size. The flow rate was monitored before and after the sampling through a portable field flowmeter kit (SKC field rotameter; 0.4–5.0 L/min).

The same PVC filters were used as field blanks for both inhalable and respirable measurements. We prepared and brought them not less than three per yard (three for respirable dust sampling and three for inhalable dust sampling, if performed simultaneously).^19^ All the field blanks have undergone the gravimetric procedure described in the next section. According to the ISO 15767:2009,^22^ blank sampling media are exposed, as closely as possible, to the same conditions as the active sampling media, without actually drawing air through. Correction is effected by subtracting the average blank mass change from the mass change of the active collection substrates. Blanks shall be matched to the samplers used; for instance, if the sampler contains a filter within a sampling cassette that is weighed, the blank shall be the same type of filter within the same type of cassette.

In order to assess the personal exposure to the combined chemical pollutants and comparing our results with the TLV‐TWA exposure limits, we referred to the European Standard EN 689/2018.^23^ In case of several hazardous chemical exposures, the risk must be assessed for all the chemical agents in combination. For this to be possible, in addition to inhalable dust, respirable dust, and RCS single exposure limits comparison, we also applied a simultaneous exposure index (*I_E_*).^24^ This index is the sum of the exposures to each airborne pollutant divided by its OELv. We applied it because a synergic action between RCS and inorganic dust cannot be excluded^6^; with regard to the inhalable dust samples, they include several dust fractions so we can consider them more dangerous than respirable dust but interrelated. We calculated the *I_E_* from the arithmetic mean (AM) exposures to the three airborne pollutants for the “all tasks.” We applied the following formula:

$$I_{E}=\sum_{i=1}^{n}\frac{E_{i}}{{\text{OELV}}_{i}}=\frac{E_{1}}{{\text{OELV}}_{1}}+\frac{E_{2}}{{\text{OELV}}_{2}}+\frac{E_{3}}{{\text{OELV}}_{3}}.$$

where *n* is the number of chemical pollutants, the worker is exposed to during the work shift and *E_i_* is the exposure to all components in the workplace atmosphere. The OELV which must be applied to the simultaneous index is 1: exceeding this limit indicates a relevant exposure to chemical risk.

### 2.1 Inhalable and respirable dust

The concentration of inhalable and respirable dust was measured by personal sampling over a full 4 to 8 h shift. The selective samplers were set in the individual breathing zone of the workers and each measurement of inhalable and respirable dust was monitored and adjusted by using field blanks. I.O.M samplers were used by setting a 2 L/min flow rate. GS‐3 Cyclones were used by setting a 2.75 L/min flow rate.

Samples and samplers carriage was performed using two De Walt TSTAK‐reinforced toolboxes equipped with pre‐cut sponges. The samples were transported by keeping them in an upright position and inside the cassettes used for the sampling. The sampling accessories were transported using a simple wheelie bin.

Gravimetric determination was performed to quantify the inhalable and respirable dust concentrations. According to the NIOSH manual of analytical methods,^20^ we used the same electronic analytical balance (0,001 mg sensitivity) before and after sample collection. The limit of detection (LOD) was calculated as three times the standard deviation of the blanks (0.035 mg). All filters were placed in suitable containers and equilibrated in a balance room at 20°C (±1°C) and 50% of relative humidity (RH) (±5% RH) for 48 h.^25^ This operation was performed before and after each measurement. For inhalable dust samples, the filter cassettes were also weighted to avoid errors due to deposition on filter holder inner walls.^26^, ^27^ For respirable dust samples, the gravimetric analyses were performed by weighting the PVC filter without the cassette.^28^

### 2.2 Respirable crystalline silica

The concentration of Respirable crystalline silica was measured through a 4‐ to 8‐h full‐shift personal air sampling, using GS‐3 Cyclones with a cut‐point of 4 µm, and 25‐mm diameter, 5‐µm pore size, PVC filters. Flow rate at 2.75 L/min was set and monitored through the portable field flowmeter before and after each sampling. The filters were, therefore, collected and analyzed by an accredited laboratory in compliance with the Unichim (Association for Unification in the Sector of Chemical Industry) M.U. 2398:11 method. The X‐ray diffraction was performed with a Philips PW3830 X‐ray generator, a Philips PW1820 goniometer, and a Philips PW1710 diffraction control unit. The calibration curve was prepared with PVC filters provided by the laboratory (deposition and weighing procedure). Quartz determination was performed by X‐ray diffraction, positioning the filter directly inside the analytical tool. A certified sample analysis and a filed blank analysis were conducted. Calibration standards were prepared by the closed chamber sampling with 99% pure quartz (with a chemical and particle‐size determination). Filters were weighted with a five decimal places scale. The LOD was calculated as <0.002 mg/m^3^.

### 2.3 Data analysis

Data were analyzed using the STATA software package (STATA analysis and statistical software, release IC/14).

The average exposure over an 8‐h time period (normal work shift), 8‐h TWA was calculated as:$\text{8 - h TWA}\;=\;\sum_{i=1}^{n}C_{i}T_{i}/8\;h$, where *C_i_* is the concentration during the *i*th interval, and *T_i_* is the duration of the *i*th interval.

The dust exposure levels were described by arithmetic mean (AM), geometric mean (GM), and geometric standard deviation (GSD).

## 3 RESULTS

Measurement time ranged from 4 to 8 h based on the duration of the different work tasks. We collected 91 dust samples. As stated in the “Methods” section, we applied a simultaneous exposure index to assess the chemical exposure to inhalable dust, respirable dust, and RCS dust for “All tasks.” Table 3 illustrates that workers involved in the selected building sites are exposed to a too high dust level (*I_E_* = 1.66) (the European Standard^23^ set an exposure limit of 1 for simultaneous exposures).

**TABLE 3:** Calculation of the simultaneous exposure index for "All tasks" for inhalable, respirable, and RCS dusts

Chemical agent	Agent (i)	OELV 8h mg/m^3^	Results (TWA) mg/m^3^ (all work tasks) (E_i_)	$\frac{E_{i}}{{\text{OELV}}_{i}}$
Inahalable dust	1	10	12.08	1.21
Respirable dust	2	3	0.88	0.29
RCS	3	0.025	0.004	0.16
Simultaneous Exposure Index (*I_E_*)				1.66

According to the EN 689,^23^ we tested the work‐related exposure compliance with the TLVs. Our findings show that due to the high workplace variability and typical exposure discontinuity in the construction industry, there is no compliance with TLVs for the work‐related groups we studied.

### 3.1 Inhalable dust

The “All tasks” geometric mean concentration of inhalable dust was 4.73 mg/m^3^, ranging from 0.01 to 90.93 mg/m^3^. A geometric mean value exceeding the 10 mg/m^3^ TWA limit value was recorded for the “manual demolition worker” group. For this group, we observed that nearly 60% of the time‐weighted measurements exceeded the ACGIH TLV‐TWA. More than the 36% of samples collected in bricklaying or similar activities have been shown to exceed the limits. For “Scaffolder and carpenter” and “Other work tasks” groups this percentage was lower. Out of 47 measurements of inhalable dust, 2 were below the level of detection. We tested the differences between inhalable dust exposed groups using an ANOVA test. We observed a statistically significant difference among the work‐related groups with regard to the inhalable dust exposure (*p* < .05). The analysis of variance among the work‐related groups with regard to the respirable dust exposure has not been statistically significant. Table 4 shows our findings.

**TABLE 4:** Concentration of inhalable dust by work task

Work task	Scaffolder and carpenter	Manual demolition worker	Bricklayer and similar	Other work tasks	All tasks
Measurements	7	12	19	9	47
Measurements <LOD	0	0	0	2	2
Average sampling time (min) (min‐max sampling time: 240–480)	308	385	320	360	343
GM mg/m^3^	2.23	13.92	5.08	1.73	4.73
GSD	3.23	3.66	2.79	11.27	5.02
AM mg/m^3^	4.57	26.56	8.69	5.80	12.08
SD	7.44	28.74	11.03	5.40	18.25
Min ‐ Max	0.42 – 21.35	1.86 – 90.93	1.14 – 46.35	0.03 – 14.48	0.01 – 90.93
% ≥ TLV	14.28	58.33	36.84	22.22	36.17

Abbreviations: AM: Arithmetic mean of the exposure concentration; LOD, Level of detection; GM, Geometric mean of the exposure concentration; GSD, Geometric Standard Deviation; SD, Standard deviation; %>TLV, percentage of exposures exceeding the TLV.

### 3.2 Respirable dust

The “All tasks” geometric mean concentration of respirable dust was 0.25 mg/m^3^, ranging from 0.015 to 9.1 mg/m^3^. No work task‐related group recorded a geometric mean higher than the ACGIH threshold limit value of 3 mg/m^3^. Apart from the “Other work tasks” group (which does not include TLV exceeding measurements), we observed that about 10% of the time‐weighted measurements exceeded the ACGIH TLV‐TWA in the remaining three groups. Out of 44 measurements of respirable dust concentrations, 9 were below the level of detection. We tested the differences between respirable dust exposed groups using a Student t test. We did not observe a statistically significant difference between the TWA respirable dust concentration of any group. Table 5 shows our findings.

**TABLE 5:** Concentration of respirable dust by work task

Work task	Scaffolder and carpenter	Manual demolition worker	Bricklayer and similar	Other work tasks	All tasks
Measurements	9	7	20	8	44
Measurements <LOD	3	1	2	3	9
Average sampling time (min) (min‐max sampling time: 240–480)	286	343	324	390	331
GM mg/m^3^	0.16	0.34	0.34	0.14	0.25
GSD	5.36	6.25	4.43	7.02	5.23
AM mg/m^3^	0.55	1.12	1.03	0.50	0.88
SD	0.97	1.97	2.10	0.72	1.70
Min ‐ Max	0.026–3.01	0.031–5.86	0.025–9.10	0.015–2.00	0.015–9.1
%≥TLV	11.11	12.5	10.00	0	9.09

Abbreviations: AM, Arithmetic mean of the exposure concentration; LOD, Level of detection; GM, Geometric mean of the exposure concentration; GSD, Geometric Standard Deviation; SD, Standard deviation; %>TLV, percentage of exposures exceeding the TLV.

### 3.3 Respirable crystalline silica

We collected 44 filters used for respirable dust sampling and we analyzed 22 to identify the RCS concentration. Out of 22 measurements, 8 were below the level of detection. In all cases, the concentration of RCS was below the threshold limit value of 0.025 mg/m^3^ recommended by the ACGIH. Table 6 shows the work‐related average concentration values.

**TABLE 6:** Average concentrations of RCS dust samples by work task

Work task	Average concentration of RCS (mg/m³)	Number of samples collected	Measurements < LOD
Manual demolition worker	0.0038	4	2
Scaffolder and carpenter	0.0057	5	1
Bricklayer and similar work tasks	0.004	10	5
Other work tasks	0.0035	3	0
All tasks	0.004	22	8

## 4 DISCUSSION AND CONCLUSIONS

The airborne inhalable dust geometric mean values, expressed as a concentration in mg/m^3^, are job‐type related and are influenced by the work tasks carried out by workers (as statistically shown in the “inhalable dust” paragraph of the Results section). As reported in Table 4, manual demolition workers inhalable dust exposure results to be exceeding the ACGIH threshold limit value. On the contrary, the average concentrations of respirable dust were found lower than the threshold limit value recommended by the above‐mentioned American association (Table 5). In order to assess an “overall” exposure taking into account all the pollutants analyzed in this study, we performed a simultaneous exposure assessment. To achieve this goal, we referred to the BS EN 689/2018^23^ and we calculated the simultaneous exposure index *I_E_*. This approach allowed us to identify an “over‐the‐limit” exposure to inhalable, respirable, and RCS dust among the “All tasks” employed in the yards.

What we have just stated can be summarized as follows:

1. The task‐specific personal dust (inhalable, respirable, RCS) sampling we performed in the post‐earthquake rebuilding sites of the city of L’Aquila show a low‐level exposure (except for demolition workers inhalable dust exposure);
2. The “overall” exposure (inhalable, respirable, RCS) assessment to dust show a global (all tasks) limit‐exceeding exposure.

In order to contextualize what we earlier stated, we examined similar studies in Scientific Literature. Our findings can be partially compared with those reported by Kirkeskov L.^5^ Both studies performed personal air sampling among two specific work tasks: “manual demolition workers” and “carpenters” (but actually our study involves 4 specific work tasks, as stated in the “methods” section). The comparison of the results shows that manual demolition workers are exposed to considerably high levels of inhalable dust. Conversely, exposure levels to respirable dust among the same kind of workers were shown to be lower than the threshold limit value. In contrast, both studies show that carpenters are exposed to inhalable and respirable dust values lower than TLVs.

Clyde Zhengdao Li et al. investigated respirable dust and RCS exposure by personal full‐shift sampling in Hong Kong construction sites.^29^ They performed 723 personal samplings and carried out analysis for respirable dust and respirable crystalline silica exposure assessment. Their findings can be globally compared with ours because they found, for all samples, low levels of respirable dust (GM 0.314 mg/m^3^) and RCS (GM 0.003 mg/m^3^). Out of the 723 measures, they found 14 samples exceeding the OSHA PEL for respirable dust and 78 samples exceeding the NIOSH REL for RCS. They referred to more permissible exposure limits compared to us: OSHA PEL (5 mg/m^3^) for RD and to NIOSH REL (0.05 mg/m^3^) for quartz.

Mohammad Normohammadi et al. determined occupational exposure to RCS by sampling in 4 construction sites in Teheran.^30^ They performed 60 personal measurements (15 per site) during building demolition activities (the job task is “demolition worker”). The results show a high personal exposure to silica (GM 0.132 mg/m^3^) which exceed the ACGIH TLV of 0.025 mg/m^3^. We found a comparable Danish study which we have already mentioned^5^: An exceeding personal concentration of RCS was found for demolition tasks (GM 0.12 mg/m^3^) in construction industry activities. As already shown in the results section of the paper, we found values about 7 times lower than the American TLV in all 4 work tasks we investigated.

About personal inhalable dust exposure during demolition activities, the Teheran study^30^ shows the concentration values exceeding the OEL. Our results and the Kirkeskov L.^5^ ones also show overtaken inhalable dust limits: Demolition workers are exposed to high levels of airborne inhalable dusts (Teheran study AM 13.37 mg/m^3^; Denmark study AM 108 mg/m^3^, GM 22.3 mg/m^3^; our study AM 26.56 mg/m^3^, GM 13.92 mg/m^3^).

The weakness of our study is the lack of qualitative analysis of air pollution. Considering the amount of building sites and of workers involved in the city of L'Aquila, it would be interesting to investigate which substances threaten workers' health. Another weakness is the limited number of measurements (especially RCS sampling). In conclusion, we must highlight that the threshold limit values we applied in this study are recommended for indoor work environments and that construction sites may have different ventilation characteristics compared to indoor work environments because of the “work in progress” status of the buildings.

The several constructions sites which have been erected across the province of L'Aquila after 2009 are subject to strict health and safety measures and inspections. Our findings are comparable with those reported in the literature although, to the best of our knowledge, at the time of this study, no other Italian comparable studies have been published.

## DISCLOSURE

*Ethical approval*: N/A. *Informed consent*: N/A. *Registry and the Registration No*. *of the study*/*Trial*: N/A. *Animal studies*: N/A. *Conflict of interest*: Authors declare no Conflict of Interests for this article.

## AUTHOR CONTRIBUTIONS

All the authors read and approved the manuscript. RM planned and carried out the sampling activities, participated in the gravimetric analyses of the dust samples, and collected and analyzed the results; AC and TL carried out the gravimetric analyses of the dust samples; EI planned and carried out the sampling activities and participated in the gravimetric analyses of the dust samples; ES managed the funding providing by the National Institute for Insurance Against Industrial Injuries and supervised the scientific activities; DP managed the funding providing by the Local Health Authority of Abruzzo and supervised the scientific activities; LC managed the funding providing by the Ente scuola edile – Comitato paritetico territoriale and supervised the scientific activities; MS collected and analyzed the results; LF managed the funding provided by the Department of Life, Health & Environmental Sciences and supervised the scientific activities.

## PREPRINT

https://www.researchsquare.com/article/rs‐7675/v1

## Data Availability

The data that support the findings of this study are available from the corresponding author upon reasonable request.
